# *N*-(4-Acetyl-3-methyl-1-phenyl-1*H*-pyrazol-5-yl)-*N*-methyl-2-(2-methyl-4-oxo-3,4-dihydroquinazolin-3-yl)benzamide

**DOI:** 10.1107/S1600536813025683

**Published:** 2013-09-25

**Authors:** Fiorella Meneghetti, Benedetta Maggio

**Affiliations:** a Department of Pharmaceutical Sciences, University of Milano, via L. Mangiagalli, 25, 20133-Milano, Italy; b Dipartimento di Scienze e Tecnologie Biologiche, Chimiche e Farmaceutiche, University of Palermo, via Archirafi, 32, 90123-Palermo, Italy

## Abstract

In the title compound, C_29_H_25_N_5_O_3_, the dihedral angle between the benzene ring and the pendant quinazoline ring system (r.m.s. deviation = 0.036Å) is 87.60 (17)°. The equivalent angle between the pyrazole ring and the phenyl group is 70.0 (2)°. The dihedral angle between the benzene and pyrazole rings is 30.7 (2)° and overall, the mol­ecular conformation approximates to a *Z* shape. A short intra­molecular C—H⋯O contact occurs. In the crystal, the mol­ecules are linked by Cπ—H⋯O-type hydrogen bonds and aromatic π–π stacking inter­actions [centroid–centroid distance = 3.860 (3) Å], generating a three-dimensional network.

## Related literature

For background to the bioactivity of methaqua­lone and its derivatives, see: Ionescu-Pioggia *et al.* (1988) ▶; Wolfe *et al.* (1990 ▶). For structural and mol­ecular modeling studies of quinazolinone derivatives, see: Duke & Codding (1993 ▶). For further synthetic details, see: Plescia *et al.* (1978 ▶).
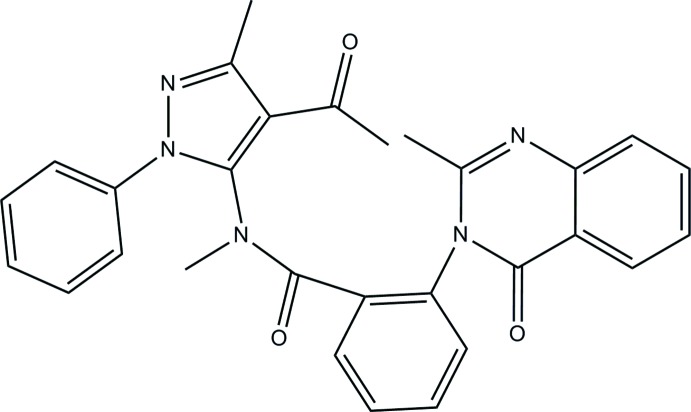


## Experimental

### 

#### Crystal data

C_29_H_25_N_5_O_3_
*M* *_r_* = 491.54
Monoclinic, 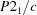

*a* = 8.617 (4) Å
*b* = 20.438 (5) Å
*c* = 15.038 (5) Å
β = 106.27 (2)°
*V* = 2542.3 (16) Å^3^
*Z* = 4
Mo *K*α radiation
μ = 0.09 mm^−1^
*T* = 293 K
0.6 × 0.5 × 0.4 mm

#### Data collection

Enraf–Nonius TurboCAD-4 diffractometer
5236 measured reflections
4401 independent reflections
1358 reflections with *I* > 2σ(*I*)
*R*_int_ = 0.062
3 standard reflections every 120 min intensity decay: −3%

#### Refinement

*R*[*F* ^2^ > 2σ(*F* ^2^)] = 0.047
*wR*(*F* ^2^) = 0.120
*S* = 0.92
4401 reflections
338 parameters
H-atom parameters constrained
Δρ_max_ = 0.16 e Å^−3^
Δρ_min_ = −0.16 e Å^−3^

### 

Data collection: *CAD-4 EXPRESS* (Enraf–Nonius, 1994 ▶); cell refinement: *CAD-4 EXPRESS*; data reduction: *XCAD4* (Harms & Wocadlo, 1995 ▶); program(s) used to solve structure: *SIR92* (Altomare *et al.*, 1994 ▶); program(s) used to refine structure: *SHELXL97* (Sheldrick, 2008 ▶); molecular graphics: *ORTEP-3 for Windows* (Farrugia, 2012 ▶); software used to prepare material for publication: *WinGX* publication routines (Farrugia, 2012 ▶).

## Supplementary Material

Crystal structure: contains datablock(s) I, global. DOI: 10.1107/S1600536813025683/hb7092sup1.cif

Structure factors: contains datablock(s) I. DOI: 10.1107/S1600536813025683/hb7092Isup2.hkl

Supplementary material file. DOI: 10.1107/S1600536813025683/hb7092Isup3.cml

Additional supplementary materials:  crystallographic information; 3D view; checkCIF report

## Figures and Tables

**Table 1:** Hydrogen-bond geometry (Å, °)

*D*—H⋯*A*	*D*—H	H⋯*A*	*D*⋯*A*	*D*—H⋯*A*
C21—H21*C*⋯O1	0.96	2.57	3.214 (5)	124
C3—H3⋯O2^i^	0.93	2.54	3.351 (6)	146
C5—H5⋯O1^ii^	0.93	2.40	3.276 (5)	157
C16—H16⋯O3^iii^	0.93	2.52	3.305 (6)	143

Symmetry codes: (i) 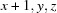; (ii) 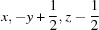; (iii) 
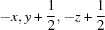
.

**Fig. 1.**
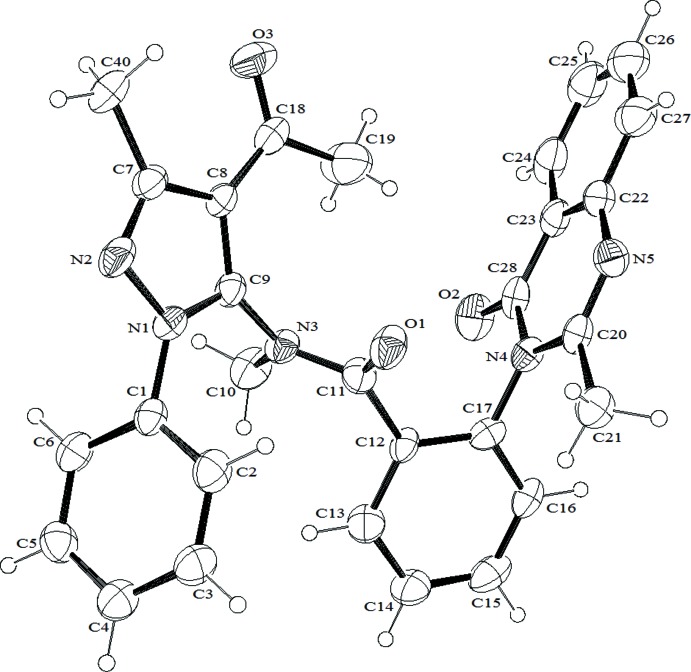
The molecular structure of the title compound, showing displacement ellipsoids for non-H atoms at the 40% probability level.

**Fig. 2.**
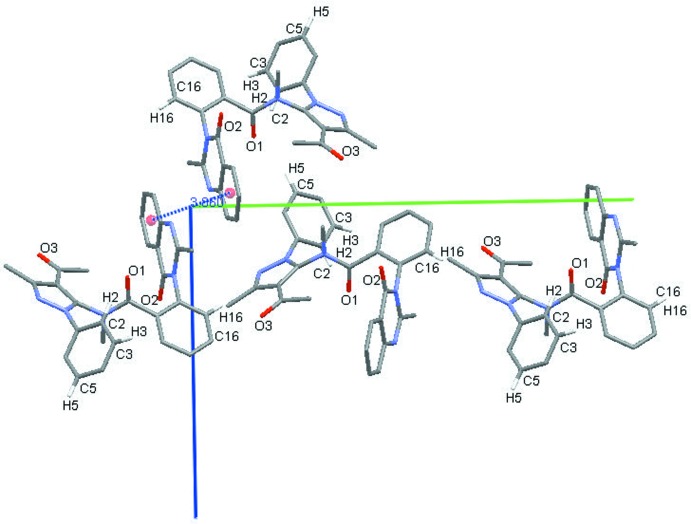
Intermolecular interactions of the title compound, viewed along the a axis. Hydrogen bonds are shown as dashed lines. H atoms not involved in hydrogen bonding have been omitted.

## supplementary crystallographic information

### 1. Comment

The product obtained from 2-acetamido-*N*-methyl-N-(3-methyl-5-phenyl-1*H*-pyrazol-5-yl)benzamide by the Bischler–Napieralski reaction (Plescia *et al.*, 1978) was hydrolized by 6 N aqueous hydrochloric acid to give the metaqualone derivative 1, whose definitive structure is now reported (Fig. 1 and 2). The compound C29 H25 N5 O3 crystallizes in the monoclinic P2_1_/c space group. The overall molecular conformation has about a *Z* shape (Fig. 2). The presence of an intramolecular hydrogen bond between C2—H2···O1 at a distance of 2.65 (1) Å, angle 129 (1)° contributes to stabilize the folded conformation of the molecule. The 2-methyl-4-oxoquinazolin-3(4*H*)-yl)benzamide moiety is characterized by an almost planar conformation, with the maximum deviation out of its best mean plane for O2 and C21 atoms by 0.103 (4) Å and -0.198 (5) Å, respectively. The bicyclic system is nearly perpendicularly oriented with respect to the N4-attached phenyl ring (dihedral angle 87.60 (17)°), while it forms with the distal ones a dihedral angle of 41.0 (1)°. The pyrazole is inclined of 70.0 (2)° with respect to both the bicyclic moiety and the C1—C6 benzene, while it presents a dihedral angle of 30.7 (2)° with the C12—C17 phenyl ring. The two benzene are oriented each other at 51.6 (2)°. The molecular packing is stabilized by intermolecular interactions type Cπ—H···O between: C3—H3···O2i of 2.51 (3) Å and 146 (1)° [symmetry code: (i) *x* + 1, *y*, *z*], C5—H5···O1ii contact of 2.40 (3) Å and 157 (1)° [symmetry code: (ii) *x*, 1/2 - *y*, *z* - 1/2], and C16—H16···O3iii at distance of 2.51 (4) Å, angle 143 (1)° [symmetry code: (iii) 2 - *x*, *y* + 1/2, 1/2 - *z*] (Fig. 3). Stacking interactions between the benzene o f the oxoquinazoline systems [centroid-centroid distance = 3.860 (3) Å] further consolidate the packing.

### 2. Experimental

A solution of the product obtained from 2-acetamido-*N*-methyl-*N*-(3-methyl-5-phenyl-1*H*-pyrazol-5-yl)benzamide by the Bischler-Napieralski reaction (Plescia *et al.*, 1978) (6 g.) in 60 ml of aqueous 6 N hydrochloric acid was refluxed for 25 minutes. The precipitated solid (3.2 g) was crystallized from ethanol-diethyl ether to give a product which was dissolved in chloroform (100 ml) and treated with triethylamine (5 ml). The solution was stirred for 1 h at room temperature, washed with water (3 × 100 ml) and dried (sodium sulfate). Removal of the solvent and the crystallization from ethanol of the residue afforded to the title compound.

### 3. Refinement

Hydrogen atoms were located by difference Fourier synthesis, except methyl and phenyl hydrogen atoms, that were introduced at calculated positions, in their described geometries and allowed to ride on the attached carbon atom with fixed isotropic thermal parameters 1.2Ueq and 1.5Ueq of the parent carbon atom for aromatic H-atoms and methyl-bound H-atoms, respectively. The crystal contains small solvent accessible voids, however, no electron density peaks were found in chemically sensible positions for solvent molecules.

### Crystal data

**Table:**

C_29_H_25_N_5_O_3_	*F*(000) = 1032
*M**_r_* = 491.54	*D*_x_ = 1.284 Mg m^−^^3^
Monoclinic, *P*2_1_/*c*	Mo *K*α radiation, λ = 0.71073 Å
Hall symbol: -P 2ybc	Cell parameters from 25 reflections
*a* = 8.617 (4) Å	θ = 9–10°
*b* = 20.438 (5) Å	µ = 0.09 mm^−^^1^
*c* = 15.038 (5) Å	*T* = 293 K
β = 106.27 (2)°	Prism, colorless
*V* = 2542.3 (16) Å^3^	0.6 × 0.5 × 0.4 mm
*Z* = 4	

### Data collection

**Table:**

Enraf–Nonius TurboCAD-4 diffractometer	*R*_int_ = 0.062
Radiation source: fine-focus sealed tube	θ_max_ = 24.9°, θ_min_ = 2.4°
Graphite monochromator	*h* = −10→9
non–profiled ω/2θ scans	*k* = −1→24
5236 measured reflections	*l* = −1→17
4401 independent reflections	3 standard reflections every 120 min
1358 reflections with *I* > 2σ(*I*)	intensity decay: −3%

### Refinement

**Table:**

Refinement on *F*^2^	0 restraints
Least-squares matrix: full	H-atom parameters constrained
*R*[*F*^2^ > 2σ(*F*^2^)] = 0.047	*w* = 1/[σ^2^(*F*_o_^2^) + (0.0352*P*)^2^] where *P* = (*F*_o_^2^ + 2*F*_c_^2^)/3
*wR*(*F*^2^) = 0.120	(Δ/σ)_max_ = 0.003
*S* = 0.92	Δρ_max_ = 0.16 e Å^−^^3^
4401 reflections	Δρ_min_ = −0.16 e Å^−^^3^
338 parameters	

### Special details

**Table:**

Geometry. All e.s.d.'s (except the e.s.d. in the dihedral angle between two l.s. planes) are estimated using the full covariance matrix. The cell e.s.d.'s are taken into account individually in the estimation of e.s.d.'s in distances, angles and torsion angles; correlations between e.s.d.'s in cell parameters are only used when they are defined by crystal symmetry. An approximate (isotropic) treatment of cell e.s.d.'s is used for estimating e.s.d.'s involving l.s. planes.
Refinement. Refinement of *F*^2^ against ALL reflections. The weighted *R*-factor *wR* and goodness of fit *S* are based on *F*^2^, conventional *R*-factors *R* are based on *F*, with *F* set to zero for negative *F*^2^. The threshold expression of *F*^2^ > σ(*F*^2^) is used only for calculating *R*-factors(gt) *etc*. and is not relevant to the choice of reflections for refinement. *R*-factors based on *F*^2^ are statistically about twice as large as those based on *F*, and *R*- factors based on ALL data will be even larger.

### Fractional atomic coordinates and isotropic or equivalent isotropic displacement parameters (Å^2^)

**Table:**

	*x*	*y*	*z*	*U*_iso_*/*U*_eq_	
C28	−0.0045 (5)	0.4366 (2)	0.2801 (3)	0.0519 (12)	
O1	0.3884 (3)	0.35430 (13)	0.2742 (2)	0.0663 (9)	
O3	0.0937 (4)	0.15749 (16)	0.3520 (2)	0.0943 (11)	
N4	0.1492 (4)	0.45942 (15)	0.2812 (2)	0.0441 (9)	
N3	0.2159 (4)	0.29993 (16)	0.1566 (2)	0.0481 (9)	
N2	0.4505 (4)	0.15359 (16)	0.2105 (2)	0.0594 (10)	
N1	0.4060 (4)	0.21360 (16)	0.1706 (2)	0.0509 (9)	
N5	0.2572 (4)	0.45536 (16)	0.4443 (2)	0.0538 (9)	
C9	0.2816 (5)	0.23970 (19)	0.1957 (3)	0.0466 (11)	
C16	0.1457 (5)	0.5305 (2)	0.1520 (3)	0.0548 (12)	
H16	0.1038	0.5639	0.1805	0.066*	
C1	0.4832 (5)	0.2402 (2)	0.1058 (3)	0.0460 (11)	
C23	−0.0249 (6)	0.42417 (19)	0.3709 (3)	0.0523 (12)	
C17	0.1780 (5)	0.4706 (2)	0.1939 (3)	0.0484 (11)	
C20	0.2709 (5)	0.46966 (18)	0.3641 (3)	0.0477 (11)	
C8	0.2412 (5)	0.19578 (19)	0.2549 (3)	0.0479 (11)	
O2	−0.1073 (3)	0.42729 (14)	0.2066 (2)	0.0772 (10)	
C10	0.0791 (5)	0.29730 (19)	0.0725 (3)	0.0685 (13)	
H10A	0.014	0.3359	0.0689	0.103*	
H10B	0.0148	0.2593	0.0745	0.103*	
H10C	0.1191	0.295	0.019	0.103*	
C40	0.3648 (5)	0.08126 (19)	0.3164 (3)	0.0791 (15)	
H40A	0.4463	0.0537	0.3036	0.119*	
H40B	0.263	0.0587	0.2997	0.119*	
H40C	0.3942	0.0917	0.3812	0.119*	
C12	0.2366 (5)	0.42010 (18)	0.1508 (3)	0.0450 (11)	
C11	0.2842 (5)	0.3564 (2)	0.1997 (3)	0.0510 (12)	
C4	0.6172 (5)	0.2944 (2)	−0.0221 (3)	0.0662 (13)	
H4	0.6613	0.3129	−0.066	0.079*	
C7	0.3506 (5)	0.1433 (2)	0.2608 (3)	0.0515 (11)	
C5	0.5239 (6)	0.2388 (2)	−0.0431 (3)	0.0622 (13)	
H5	0.5063	0.2196	−0.1011	0.075*	
C3	0.6448 (5)	0.3221 (2)	0.0637 (4)	0.0682 (13)	
H3	0.7082	0.3596	0.0781	0.082*	
C21	0.4242 (5)	0.50044 (19)	0.3564 (3)	0.0629 (13)	
H21A	0.4986	0.5048	0.417	0.094*	
H21B	0.401	0.5429	0.3285	0.094*	
H21C	0.4714	0.4734	0.3187	0.094*	
C13	0.2639 (5)	0.4318 (2)	0.0658 (3)	0.0611 (12)	
H13	0.3029	0.3983	0.036	0.073*	
C22	0.1086 (6)	0.4322 (2)	0.4488 (3)	0.0531 (11)	
C2	0.5792 (5)	0.2949 (2)	0.1288 (3)	0.0600 (12)	
H2	0.5996	0.3132	0.1876	0.072*	
C24	−0.1740 (6)	0.4032 (2)	0.3805 (4)	0.0768 (15)	
H24	−0.2624	0.3981	0.3288	0.092*	
C26	−0.0550 (8)	0.3968 (2)	0.5449 (4)	0.0877 (17)	
H26	−0.0652	0.3871	0.6034	0.105*	
C15	0.1749 (5)	0.5416 (2)	0.0680 (3)	0.0697 (14)	
H15	0.1544	0.5826	0.0405	0.084*	
C6	0.4571 (5)	0.2117 (2)	0.0202 (3)	0.0591 (12)	
H6	0.3942	0.1741	0.0056	0.071*	
C14	0.2338 (5)	0.4928 (2)	0.0249 (3)	0.0660 (13)	
H14	0.2536	0.5006	−0.0319	0.079*	
C18	0.1174 (5)	0.2017 (2)	0.3040 (3)	0.0612 (13)	
C25	−0.1877 (7)	0.3903 (2)	0.4688 (5)	0.0877 (18)	
H25	−0.2864	0.3772	0.4765	0.105*	
C19	0.0256 (6)	0.2638 (2)	0.3005 (4)	0.113 (2)	
H19A	−0.0472	0.2597	0.3382	0.17*	
H19B	−0.0349	0.2728	0.2377	0.17*	
H19C	0.0997	0.2991	0.3234	0.17*	
C27	0.0913 (6)	0.4173 (2)	0.5363 (4)	0.0730 (15)	
H27	0.1791	0.4213	0.5886	0.088*	

### Atomic displacement parameters (Å^2^)

**Table:**

	*U*^11^	*U*^22^	*U*^33^	*U*^12^	*U*^13^	*U*^23^
C28	0.045 (3)	0.040 (3)	0.064 (3)	0.001 (2)	0.003 (3)	−0.015 (3)
O1	0.085 (2)	0.0449 (19)	0.056 (2)	0.0109 (16)	−0.0015 (18)	0.0038 (16)
O3	0.121 (3)	0.063 (2)	0.118 (3)	−0.003 (2)	0.065 (2)	0.020 (2)
N4	0.034 (2)	0.042 (2)	0.049 (2)	−0.0039 (17)	−0.001 (2)	−0.0022 (18)
N3	0.049 (2)	0.041 (2)	0.048 (2)	0.0066 (19)	0.0030 (18)	0.007 (2)
N2	0.072 (3)	0.037 (2)	0.068 (3)	0.011 (2)	0.017 (2)	0.009 (2)
N1	0.061 (2)	0.036 (2)	0.057 (2)	0.005 (2)	0.018 (2)	0.0066 (19)
N5	0.050 (2)	0.058 (2)	0.053 (2)	0.000 (2)	0.013 (2)	−0.004 (2)
C9	0.054 (3)	0.036 (3)	0.048 (3)	0.009 (2)	0.011 (2)	0.003 (2)
C16	0.059 (3)	0.030 (3)	0.068 (3)	0.003 (2)	0.005 (3)	0.001 (3)
C1	0.050 (3)	0.038 (3)	0.046 (3)	0.004 (2)	0.008 (2)	−0.003 (2)
C23	0.050 (3)	0.040 (3)	0.070 (4)	−0.001 (2)	0.021 (3)	−0.010 (2)
C17	0.044 (3)	0.044 (3)	0.050 (3)	0.000 (2)	0.001 (2)	0.010 (2)
C20	0.039 (3)	0.037 (3)	0.063 (3)	0.004 (2)	0.007 (3)	−0.007 (3)
C8	0.058 (3)	0.035 (3)	0.051 (3)	0.006 (2)	0.015 (2)	0.004 (2)
O2	0.055 (2)	0.084 (2)	0.079 (2)	−0.0142 (17)	−0.0042 (19)	−0.0180 (19)
C10	0.067 (3)	0.061 (3)	0.062 (3)	0.007 (3)	−0.007 (3)	0.011 (3)
C40	0.105 (4)	0.038 (3)	0.093 (4)	0.012 (3)	0.026 (3)	0.012 (3)
C12	0.051 (3)	0.032 (3)	0.046 (3)	0.007 (2)	0.004 (2)	0.006 (2)
C11	0.056 (3)	0.050 (3)	0.042 (3)	0.007 (3)	0.006 (2)	0.002 (3)
C4	0.067 (3)	0.060 (3)	0.070 (4)	−0.006 (3)	0.018 (3)	0.001 (3)
C7	0.068 (3)	0.036 (3)	0.046 (3)	0.002 (3)	0.008 (2)	0.005 (2)
C5	0.073 (3)	0.060 (3)	0.051 (3)	0.001 (3)	0.012 (3)	−0.006 (3)
C3	0.064 (3)	0.059 (3)	0.084 (4)	−0.019 (2)	0.023 (3)	−0.011 (3)
C21	0.051 (3)	0.071 (3)	0.061 (3)	−0.012 (3)	0.006 (2)	−0.011 (3)
C13	0.072 (3)	0.056 (3)	0.052 (3)	0.008 (3)	0.011 (3)	0.006 (3)
C22	0.060 (3)	0.043 (3)	0.060 (3)	0.006 (2)	0.022 (3)	−0.002 (2)
C2	0.064 (3)	0.061 (3)	0.050 (3)	−0.011 (3)	0.006 (2)	−0.010 (3)
C24	0.066 (4)	0.054 (3)	0.116 (5)	−0.008 (3)	0.036 (3)	−0.027 (3)
C26	0.109 (5)	0.061 (4)	0.110 (5)	0.013 (4)	0.058 (5)	0.005 (3)
C15	0.073 (3)	0.051 (3)	0.071 (4)	−0.008 (3)	−0.003 (3)	0.018 (3)
C6	0.059 (3)	0.046 (3)	0.070 (3)	−0.012 (2)	0.015 (3)	−0.009 (3)
C14	0.081 (4)	0.059 (3)	0.053 (3)	−0.001 (3)	0.010 (3)	0.015 (3)
C18	0.074 (3)	0.035 (3)	0.074 (4)	−0.003 (3)	0.020 (3)	0.002 (3)
C25	0.083 (5)	0.064 (3)	0.135 (6)	−0.015 (3)	0.063 (5)	−0.016 (4)
C19	0.138 (5)	0.084 (4)	0.148 (5)	0.043 (4)	0.089 (4)	0.040 (4)
C27	0.078 (4)	0.060 (3)	0.084 (4)	0.011 (3)	0.027 (3)	0.005 (3)

### Geometric parameters (Å, º)

**Table:**

C28—O2	1.223 (4)	C40—H40B	0.96
C28—N4	1.400 (4)	C40—H40C	0.96
C28—C23	1.446 (5)	C12—C13	1.383 (5)
O1—C11	1.225 (4)	C12—C11	1.495 (5)
O3—C18	1.210 (4)	C4—C3	1.368 (5)
N4—C20	1.402 (4)	C4—C5	1.375 (5)
N4—C17	1.421 (4)	C4—H4	0.93
N3—C11	1.373 (5)	C5—C6	1.361 (5)
N3—C9	1.412 (4)	C5—H5	0.93
N3—C10	1.469 (4)	C3—C2	1.379 (5)
N2—C7	1.312 (4)	C3—H3	0.93
N2—N1	1.372 (4)	C21—H21A	0.96
N1—C9	1.343 (4)	C21—H21B	0.96
N1—C1	1.431 (5)	C21—H21C	0.96
N5—C20	1.278 (4)	C13—C14	1.384 (5)
N5—C22	1.385 (5)	C13—H13	0.93
C9—C8	1.376 (5)	C22—C27	1.399 (5)
C16—C17	1.370 (5)	C2—H2	0.93
C16—C15	1.374 (5)	C24—C25	1.390 (6)
C16—H16	0.93	C24—H24	0.93
C1—C6	1.372 (5)	C26—C27	1.368 (6)
C1—C2	1.377 (5)	C26—C25	1.379 (6)
C23—C24	1.399 (5)	C26—H26	0.93
C23—C22	1.403 (5)	C15—C14	1.363 (5)
C17—C12	1.387 (5)	C15—H15	0.93
C20—C21	1.497 (5)	C6—H6	0.93
C8—C7	1.414 (5)	C14—H14	0.93
C8—C18	1.462 (5)	C18—C19	1.489 (5)
C10—H10A	0.96	C25—H25	0.93
C10—H10B	0.96	C19—H19A	0.96
C10—H10C	0.96	C19—H19B	0.96
C40—C7	1.505 (5)	C19—H19C	0.96
C40—H40A	0.96	C27—H27	0.93
			
O2—C28—N4	120.4 (4)	C5—C4—H4	120.2
O2—C28—C23	125.2 (4)	N2—C7—C8	112.1 (4)
N4—C28—C23	114.4 (4)	N2—C7—C40	119.4 (4)
C28—N4—C20	122.0 (4)	C8—C7—C40	128.5 (4)
C28—N4—C17	116.9 (3)	C6—C5—C4	120.7 (4)
C20—N4—C17	121.1 (4)	C6—C5—H5	119.6
C11—N3—C9	117.9 (3)	C4—C5—H5	119.6
C11—N3—C10	124.9 (3)	C4—C3—C2	120.4 (4)
C9—N3—C10	117.2 (3)	C4—C3—H3	119.8
C7—N2—N1	104.3 (3)	C2—C3—H3	119.8
C9—N1—N2	112.3 (3)	C20—C21—H21A	109.5
C9—N1—C1	126.9 (3)	C20—C21—H21B	109.5
N2—N1—C1	120.7 (3)	H21A—C21—H21B	109.5
C20—N5—C22	117.1 (4)	C20—C21—H21C	109.5
N1—C9—C8	106.8 (3)	H21A—C21—H21C	109.5
N1—C9—N3	119.2 (4)	H21B—C21—H21C	109.5
C8—C9—N3	133.9 (4)	C12—C13—C14	120.5 (4)
C17—C16—C15	120.3 (4)	C12—C13—H13	119.7
C17—C16—H16	119.8	C14—C13—H13	119.7
C15—C16—H16	119.8	N5—C22—C27	117.7 (5)
C6—C1—C2	120.7 (4)	N5—C22—C23	123.5 (4)
C6—C1—N1	119.5 (4)	C27—C22—C23	118.8 (5)
C2—C1—N1	119.8 (4)	C1—C2—C3	119.1 (4)
C24—C23—C22	120.7 (5)	C1—C2—H2	120.5
C24—C23—C28	120.6 (5)	C3—C2—H2	120.5
C22—C23—C28	118.7 (4)	C25—C24—C23	118.9 (5)
C16—C17—C12	120.2 (4)	C25—C24—H24	120.6
C16—C17—N4	120.2 (4)	C23—C24—H24	120.6
C12—C17—N4	119.6 (4)	C27—C26—C25	121.5 (6)
N5—C20—N4	124.1 (4)	C27—C26—H26	119.2
N5—C20—C21	119.2 (4)	C25—C26—H26	119.2
N4—C20—C21	116.7 (4)	C14—C15—C16	120.4 (4)
C9—C8—C7	104.5 (4)	C14—C15—H15	119.8
C9—C8—C18	128.7 (4)	C16—C15—H15	119.8
C7—C8—C18	126.8 (4)	C5—C6—C1	119.5 (4)
N3—C10—H10A	109.5	C5—C6—H6	120.2
N3—C10—H10B	109.5	C1—C6—H6	120.2
H10A—C10—H10B	109.5	C15—C14—C13	119.7 (4)
N3—C10—H10C	109.5	C15—C14—H14	120.1
H10A—C10—H10C	109.5	C13—C14—H14	120.1
H10B—C10—H10C	109.5	O3—C18—C8	120.6 (4)
C7—C40—H40A	109.5	O3—C18—C19	118.7 (4)
C7—C40—H40B	109.5	C8—C18—C19	120.6 (4)
H40A—C40—H40B	109.5	C26—C25—C24	120.1 (5)
C7—C40—H40C	109.5	C26—C25—H25	120
H40A—C40—H40C	109.5	C24—C25—H25	120
H40B—C40—H40C	109.5	C18—C19—H19A	109.5
C13—C12—C17	118.8 (4)	C18—C19—H19B	109.5
C13—C12—C11	120.7 (4)	H19A—C19—H19B	109.5
C17—C12—C11	120.2 (4)	C18—C19—H19C	109.5
O1—C11—N3	120.6 (4)	H19A—C19—H19C	109.5
O1—C11—C12	120.7 (4)	H19B—C19—H19C	109.5
N3—C11—C12	118.5 (4)	C26—C27—C22	119.9 (5)
C3—C4—C5	119.6 (4)	C26—C27—H27	120
C3—C4—H4	120.2	C22—C27—H27	120
			
O2—C28—N4—C20	−178.9 (4)	C10—N3—C11—O1	173.4 (4)
C23—C28—N4—C20	−0.7 (5)	C9—N3—C11—C12	169.3 (4)
O2—C28—N4—C17	−0.1 (5)	C10—N3—C11—C12	−10.6 (6)
C23—C28—N4—C17	178.1 (3)	C13—C12—C11—O1	115.6 (5)
C7—N2—N1—C9	0.0 (4)	C17—C12—C11—O1	−58.4 (6)
C7—N2—N1—C1	−176.5 (4)	C13—C12—C11—N3	−60.4 (5)
N2—N1—C9—C8	0.2 (4)	C17—C12—C11—N3	125.5 (4)
C1—N1—C9—C8	176.4 (4)	N1—N2—C7—C8	−0.2 (4)
N2—N1—C9—N3	−176.7 (3)	N1—N2—C7—C40	−179.4 (3)
C1—N1—C9—N3	−0.5 (6)	C9—C8—C7—N2	0.3 (5)
C11—N3—C9—N1	−87.6 (4)	C18—C8—C7—N2	−177.8 (4)
C10—N3—C9—N1	92.3 (4)	C9—C8—C7—C40	179.4 (4)
C11—N3—C9—C8	96.5 (5)	C18—C8—C7—C40	1.2 (7)
C10—N3—C9—C8	−83.6 (6)	C3—C4—C5—C6	0.8 (6)
C9—N1—C1—C6	−106.9 (5)	C5—C4—C3—C2	−0.2 (7)
N2—N1—C1—C6	69.0 (5)	C17—C12—C13—C14	0.2 (6)
C9—N1—C1—C2	71.4 (5)	C11—C12—C13—C14	−173.9 (4)
N2—N1—C1—C2	−112.6 (4)	C20—N5—C22—C27	−179.1 (4)
O2—C28—C23—C24	−3.9 (6)	C20—N5—C22—C23	−0.3 (6)
N4—C28—C23—C24	177.9 (3)	C24—C23—C22—N5	−177.1 (4)
O2—C28—C23—C22	174.7 (4)	C28—C23—C22—N5	4.2 (6)
N4—C28—C23—C22	−3.4 (5)	C24—C23—C22—C27	1.6 (6)
C15—C16—C17—C12	−1.6 (6)	C28—C23—C22—C27	−177.1 (4)
C15—C16—C17—N4	179.4 (4)	C6—C1—C2—C3	2.2 (6)
C28—N4—C17—C16	89.6 (4)	N1—C1—C2—C3	−176.2 (4)
C20—N4—C17—C16	−91.6 (5)	C4—C3—C2—C1	−1.2 (6)
C28—N4—C17—C12	−89.4 (4)	C22—C23—C24—C25	−0.1 (6)
C20—N4—C17—C12	89.4 (5)	C28—C23—C24—C25	178.5 (4)
C22—N5—C20—N4	−4.2 (6)	C17—C16—C15—C14	1.1 (7)
C22—N5—C20—C21	174.7 (3)	C4—C5—C6—C1	0.1 (6)
C28—N4—C20—N5	4.9 (6)	C2—C1—C6—C5	−1.6 (6)
C17—N4—C20—N5	−173.9 (4)	N1—C1—C6—C5	176.8 (4)
C28—N4—C20—C21	−174.0 (3)	C16—C15—C14—C13	0.1 (7)
C17—N4—C20—C21	7.2 (5)	C12—C13—C14—C15	−0.8 (6)
N1—C9—C8—C7	−0.3 (4)	C9—C8—C18—O3	177.9 (4)
N3—C9—C8—C7	176.0 (4)	C7—C8—C18—O3	−4.3 (7)
N1—C9—C8—C18	177.8 (4)	C9—C8—C18—C19	−5.9 (7)
N3—C9—C8—C18	−5.9 (8)	C7—C8—C18—C19	171.8 (4)
C16—C17—C12—C13	1.0 (6)	C27—C26—C25—C24	1.4 (8)
N4—C17—C12—C13	180.0 (4)	C23—C24—C25—C26	−1.3 (7)
C16—C17—C12—C11	175.1 (4)	C25—C26—C27—C22	0.1 (7)
N4—C17—C12—C11	−5.9 (6)	N5—C22—C27—C26	177.3 (4)
C9—N3—C11—O1	−6.7 (5)	C23—C22—C27—C26	−1.5 (6)

### Hydrogen-bond geometry (Å, º)

**Table:**

*D*—H···*A*	*D*—H	H···*A*	*D*···*A*	*D*—H···*A*
C21—H21*C*···O1	0.96	2.57	3.214 (5)	124
C3—H3···O2^i^	0.93	2.54	3.351 (6)	146
C5—H5···O1^ii^	0.93	2.40	3.276 (5)	157
C16—H16···O3^iii^	0.93	2.52	3.305 (6)	143

Symmetry codes: (i) *x*+1, *y*, *z*; (ii) *x*, −*y*+1/2, *z*−1/2; (iii) −*x*, *y*+1/2, −*z*+1/2.

## References

[bb1] Altomare, A., Cascarano, G., Giacovazzo, C., Guagliardi, A., Burla, M. C., Polidori, G. & Camalli, M. (1994). *J. Appl. Cryst.* **27**, 435.

[bb2] Duke, N. E. C. & Codding, P. W. (1993). *Acta Cryst.* B**49**, 719–726. 10.1107/s0108768193000576 8397980

[bb3] Enraf–Nonius (1994). *CAD-4 EXPRESS* Enraf–Nonius, Delft, The Netherlands.

[bb4] Farrugia, L. J. (2012). *J. Appl. Cryst.* **45**, 849–854.

[bb5] Harms, K. & Wocadlo, S. (1995). *XCAD4* University of Marburg, Germany.

[bb6] Ionescu-Pioggia, M., Bird, M., Orzaci, M. H., Benes, F., Beake, B. & Cole, J. O. (1988). *Int. Clin. Psyco. Pharmacol.* **3**, 97–109. 10.1097/00004850-198804000-00001 3397524

[bb7] Plescia, S., Daidone, G., Sprio, V., Aiello, E., Dattolo, G. & Cirrincione, G. (1978). *J. Heterocycl. Chem.* **15**, 1339–1342.

[bb8] Sheldrick, G. M. (2008). *Acta Cryst.* A**64**, 112–122. 10.1107/S0108767307043930 18156677

[bb9] Wolfe, J. F., Rathman, T. L., Sleevi, M. C., Campbell, J. A. & Greenwood, T. D. (1990). *J. Med. Chem.* **33**, 161–166. 10.1021/jm00163a027 2296016

